# In vitro and in vivo characterization of the JAK1 selectivity of upadacitinib (ABT-494)

**DOI:** 10.1186/s41927-018-0031-x

**Published:** 2018-08-28

**Authors:** Julie M. Parmentier, Jeff Voss, Candace Graff, Annette Schwartz, Maria Argiriadi, Michael Friedman, Heidi S. Camp, Robert J. Padley, Jonathan S. George, Deborah Hyland, Matthew Rosebraugh, Neil Wishart, Lisa Olson, Andrew J. Long

**Affiliations:** 1 Immunology Discovery Research, AbbVie Bioresearch Center, 100 Research Dr, Worcester, MA 01605 USA; 2 Immunology Clinical Development, AbbVie, 1 North Waukegan Rd, North Chicago, IL 60064 USA; 3 Clinical Pharmacology and Pharmacometrics, AbbVie, North Chicago, IL USA

**Keywords:** Arthritis, rheumatoid, Selectivity, Kinase, JAK inhibitor

## Abstract

**Background:**

Anti-cytokine therapies such as adalimumab, tocilizumab, and the small molecule JAK inhibitor tofacitinib have proven that cytokines and their subsequent downstream signaling processes are important in the pathogenesis of rheumatoid arthritis. Tofacitinib, a pan-JAK inhibitor, is the first approved JAK inhibitor for the treatment of RA and has been shown to be effective in managing disease. However, in phase 2 dose-ranging studies tofacitinib was associated with dose-limiting tolerability and safety issues such as anemia. Upadacitinib (ABT-494) is a selective JAK1 inhibitor that was engineered to address the hypothesis that greater JAK1 selectivity over other JAK family members will translate into a more favorable benefit:risk profile. Upadacitinib selectively targets JAK1 dependent disease drivers such as IL-6 and IFNγ, while reducing effects on reticulocytes and natural killer (NK) cells, which potentially contributed to the tolerability issues of tofacitinib.

**Methods:**

Structure-based hypotheses were used to design the JAK1 selective inhibitor upadacitinib. JAK family selectivity was defined with in vitro assays including biochemical assessments, engineered cell lines, and cytokine stimulation. In vivo selectivity was defined by the efficacy of upadacitinib and tofacitinib in a rat adjuvant induced arthritis model, activity on reticulocyte deployment, and effect on circulating NK cells. The translation of the preclinical JAK1 selectivity was assessed in healthy volunteers using ex vivo stimulation with JAK-dependent cytokines.

**Results:**

Here, we show the structural basis for the JAK1 selectivity of upadacitinib, along with the in vitro JAK family selectivity profile and subsequent in vivo physiological consequences. Upadacitinib is ~ 60 fold selective for JAK1 over JAK2, and > 100 fold selective over JAK3 in cellular assays. While both upadacitinib and tofacitinib demonstrated efficacy in a rat model of arthritis, the increased selectivity of upadacitinib for JAK1 resulted in a reduced effect on reticulocyte deployment and NK cell depletion relative to efficacy. Ex vivo pharmacodynamic data obtained from Phase I healthy volunteers confirmed the JAK1 selectivity of upadactinib in a clinical setting.

**Conclusions:**

The data presented here highlight the JAK1 selectivity of upadacinitinib and supports its use as an effective therapy for the treatment of RA with the potential for an improved benefit:risk profile.

**Electronic supplementary material:**

The online version of this article (10.1186/s41927-018-0031-x) contains supplementary material, which is available to authorized users.

## Background

Over the last 30 years, the treatment of rheumatoid arthritis (RA) has been revolutionized with the development of biologic therapies such as anti-TNF, anti-IL-6, CTLA4-Ig and anti-CD20 agents, among others. The efficacy of these therapies has provided patients with multiple options for managing their disease. Besides providing benefit for patients, these agents have demonstrated clinical evidence of important pro-inflammatory molecular pathways that are operant in the pathogenesis of RA, including cytokine receptor signaling [1–4]. Even with the significant improvement in care, there are limitations to the maximal benefit achieved by these therapies, which may be a function of targeting a single cytokine or cell type. Based on this clinical understanding, medicines that target multiple pro-inflammatory mechanisms, such as the Janus kinase (JAK) family of intracellular signaling enzymes, are being developed for the treatment of RA.

The JAK family of enzymes (JAK1, JAK2, JAK3 and tyrosine kinase 2 (TYK2)) are important signaling molecules involved in a diverse range of biological functions such as cytokine and growth factor signaling [5, 6]. JAK molecules act cooperatively to transduce cytokine signaling but in certain cellular settings may preferentially use one JAK over another. For example, IL-6 signaling, which is important in the pathogenesis of RA, results in activation of JAK1, JAK2 and TYK2 with JAK1 playing a predominant role [7]. Erythropoietin (EPO) receptor signaling utilizes JAK2 for signal transduction and is a critical growth factor in the development and deployment of reticulocytes and red blood cells [8]. JAK3 in conjuction with JAK1 is an important component of signaling transduction for cytokine receptors that utilize the common gamma chain (γc) such as IL-2, IL-4, IL-7, IL-9, IL-15 and IL-21 [9]. These cytokines are involved in a range of physiologic processes including T cell survival, Th2 responses, and natural killer (NK) cell survival. Mice deficient in JAK3, for example, have no circulating NK cells, indicating a critical role for JAK3 in NK cell survival. In humans, autosomal recessive JAK3 deficiency manifests in a lack of T cells and NK cells, resulting in a severe combined immunodeficiency (SCID) phenotype [10].

Tofacitinib is a pan-JAK inhibitor that was the first approved JAK inhibitor for the treatment of RA and the first agent of the class to demonstrate proof of concept. In phase 2 dose ranging studies tofacitnib demonstrated dose dependent increases in efficacy with 5, 10 and 15 mg doses. However, the improved efficacy was associated with a small, but dose-dependent increase in the percentage of subjects with hemoglobin reduction that were considered severe [11]. Despite the broader kinome selectivity of tofacitinib, this molecule has limited selectivity within the JAK family of kinases [12]. Tofactinib may not have the optimal JAK family selectivity profile to fully achieve the therapeutic potential for JAK family inhibition. Based on these observations, we explored the hypothesis that compounds with increased JAK1 selectivity within the JAK family could provide similar or even increased efficacy while sparing some of the dose-limiting side effects associated with tofacitinib.

Here we describe upadacitinib, a novel JAK inhibitor engineered for JAK1 selectivity. Upadacitinib is selective against the broader kinome and demonstrates JAK family selectivity in multiple cellular assay systems. We also show the physiologic consequence of JAK1 selectivity in vivo by comparing the efficacy of upadacitinib and tofacitinib in a rat model of RA with the impact on reticulocyte deployment and circulating NK cell counts.

## Methods

### Enzyme potency and selectivity assays

Active recombinant human catalytic domains of JAK1 (aa 845–1142) and JAK3 (aa 811–1103) were prepared in house and expressed in SF9 cells as a glutathione s transferase (GST) fusion and purified by glutathione affinity chromatography. Active human TYK2 (aa880–1185) was purified in house and contains an N-terminal histidine-tag and C-terminal FLAG tag. It was purified by immobilized metal ion affinity chromatography. Recombinant kinase domain of JAK2 was purchased from Millipore (Burlington, MA). Peptides Biotin-TYR2 (Biotin-(Ahx)-AEEEYFFLFA-amide) and Biotin-TYR1 (Biotin-(Ahx)-GAEEEIYAAFFA-COOH were purchased from New England Peptide (Gardner, MA). Reactions were carried out at 100 μM ATP in the presence of inhibitor and 2 μM peptide. For competition assays, the JAK1 IC_50_ of upadacitinib was determined in the presence of varying amounts of ATP (0.01-1 mM) equal to and greater than the ATP Km for the kinase. ATP competitiveness was evaluated using the Cheng-Prusoff equation. Inhibitors that are ATP competitive will display changes in the IC_50_ consistent with the theoretical values derived from the Cheng-Prusoff equation at varying ATP concentrations.

### Ba/F3 cellular potency and selectivity assays

The TEL-JAK2, TEL-JAK3, TEL-TYK2, and BCR-JAK1 Ba/F3 engineered cell lines were purchased from Advanced Cellular Dynamics (San Diego, CA). Cells were grown in RPMI 1640 media supplemented with 10% fetal bovine serum, 1× penicillin-streptomycin-glutamine and 0.5 μg/ml puromycin (ThermoFisher Scientific, Waltham, MA).

For measurement of phosphorylation of signal transducer and activator of transcription 5 (pSTAT5), cells were washed and resuspended in Hank’s balanced salt solution at a density of 2 X 10^7^ cells/mL. Five microliters of cell suspension were added to a 384-well, low-volume, white-walled polystyrene plate (Perkin Elmer, Hopkinton, MA) that contained 5 μL of compound (in a 11 point [1:3] titration series). Cells were incubated with compound (final DMSO concentration 0.5%) for 30 min at 37 °C before proceeding with pSTAT5 detection. pSTAT5 was measured with the SureFire pSTAT5 Assay kit (Perkin Elmer, Hopkinton, MA) per standard manufacturer’s protocol, with the exception of an overnight incubation following addition of donor beads before detection on the EnVision (Perkin Elmer, Hopkinton, MA).

### Cytokine potency assays

IL-6 and Oncostatin M (OSM) induced STAT3 phosphorylation was assessed in the human erythroleukemia TF-1 cell line. Erythropoietin-induced STAT5 phosphorylation was assessed in the human UT-7 cell line. IL-2 and IL-15 induced STAT5 phosphorylation was assessed in activated human T-cells. Detection of phosphorylated STATs was accomplished with the SureFire pSTAT5 or pSTAT3 Assay kit (Perkin Elmer, Hopkinton, MA) per standard manufacturer’s protocol, with the exception of an overnight incubation following addition of donor beads before detection on the EnVision (Perkin Elmer, Hopkinton, MA). IFNγ induced STAT1 phosphorylation was assessed in the CD14+ monocyte population in human PBMC by flow cytometry. CD14 BV421 was purchased from BD Biosciences (San Jose, CA). STAT1 PE (pY705) was purchased from ThermoFisher Scientific (Waltham, MA). IL-4 and IL-13 induced STAT6 phosphorylation and IL-31 induced STAT3 phosphorylation were assessed in adult human epithelial keratinocytes (HEKa, ThermoFisher Scientific, Waltham, MA) by flow cytometry. STAT6 PE (pY641) and STAT3 PE (Y705) were purchased from BD Biosciences (San Jose, CA).

### Rat adjuvant-induced arthritis (AIA) model

Arthritis was induced in female Lewis rats (weight, 125 – 150 g) (Charles River, Portage, MI) by a single intradermal injection of 0.1 mL of microbacterium tuberculosis emulsion into the right hind footpad (Day 0). Rats were dosed as indicated orally by gavage twice a day (BID) for 10 days (Day7 – Day17) post immunization with either vehicle or study drug. To evaluate the severity of arthritis, paw swelling was evaluated with a water displacement plethysmograph (Ugo Basile North America Inc. PA) every other day up to Day 17. On Day 17, all rats were exsanguinated by cardiac puncture under isolfuorane anesthesia. Left rear paws were scanned using a μCT (SCANCO Medical, Southeastern, PA, Model #μCT40). Bone volume and density were determined in a 360 μm vertical section encompassing the tarsal section of the paw.

### Reticulocyte deployment assays

Naïve male Lewis rats were injected intravenously with either PBS or 1000 IU of epoetin α (Procrit®, Janssen Products, LP, Horsham, PA) for two consecutive days. Reticulocytes were measured on day 4 by flow cytometry using thiozole orange as a dye as previously described [13]. Dose responses of either upadacitinib or tofacitinib were dosed 30 min prior to the first Epo injection and then once every 12 h subsequently for 3 days.

### NK cell analysis

Sprague Dawley rats were dosed orally with either upadacitinib or tofacitinib at doses indicated for 14 days. Blood was collected and stained using BD MultiTest IMK kit (BD Biosciences, San Jose, CA) per manufacturer’s instructions. NK cell numbers were determined by using FlowJo analysis software (FlowJo, LLC, Ashland, OR) and by examining the CD3^−^/CD16^+^/CD56^+^ population. The number of cells/μL was calculated by using the following equation: (# events in cell population/# of events in absolute bead count region) × (# beads per test/test volume), with the value beads per test indicated on the BD Trucount tube label.

### Pharmacokinetic/pharmacodynamics modeling

A direct maximum enhancement model was the most predictive for defining the efficacious concentration range and human efficacious dose. Efficacious area under the concentration-versus-time curve (AUC) was based on paw swelling on the last day of the study plotted against the cumulative plasma concentration of upadacitinib or tofacitinib over 12 h (AUC_0–12_).

### Clinical ex vivo stimulation assays

For each subject, blood was collected by venipuncture into 2 mL sodium heparin tubes at 0, 1, 6, and 12 h post upadacitinib or tofacitinib dose. Recombinant human IL-6 (400 ng/ml), or IL-7 (400 ng/ml), (R&D Systems, Minneapolis, MN) was added to blood and incubated for 10 min at 37°C. Surface antibodies were added (CD14-APC, CD3-fluorescein isothiocyanate [FITC]; BD Biosciences, San Jose, CA) and incubated on ice for an additional 20 min. Samples were lysed (Lyse/Fix, BD Biosciences, San Jose, CA) and incubated for 10 min at 37°C. Samples were washed and stored at − 70°C. For intracellular staining, samples were thawed, washed, and resuspended in BD Perm buffer III (BD Biosciences, San Jose, CA) on ice for 30 min. Samples were washed and stained with pSTAT5-PE or pSTAT3-PE (BD Biosciences, San Jose, CA) for 60 min at room temperature and then analyzed immediately on a FACSCalibur. Geometric means were determined using FlowJo analysis software. Percent inhibition of relevant STAT phosphorylation was calculated as follows: (1-(Induction of pSTAT at 1 h – baseline pSTAT at 0 h) / (Induction of pSTAT at 0 h – baseline pSTAT at 0 h)*100.

### Statistics

In the AIA experiments, significant differences were determined by comparing each treatment group to vehicle with Dunnett’s multiple comparison test. *P* < 0.05 was considered indicative of statistically significant differences among groups.

## Results

### Design of selective JAK1 inhibitors

**Fig. 1:**
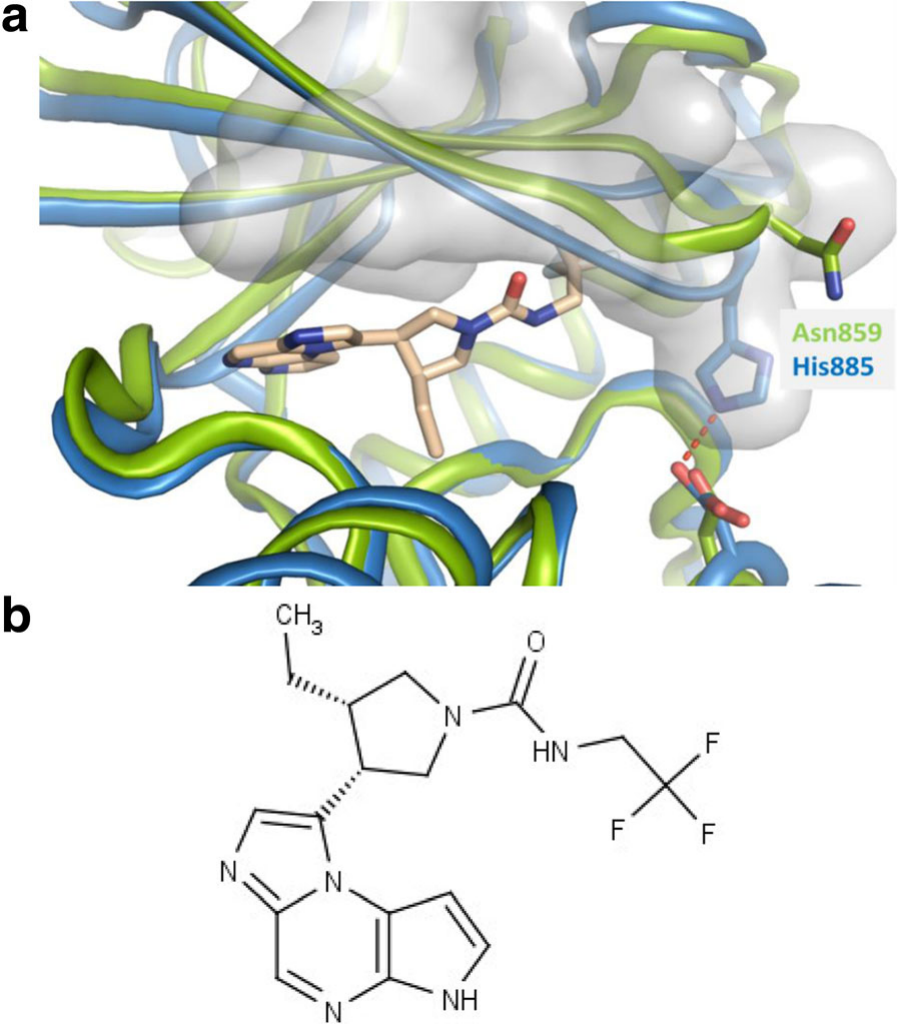
A. Upadacitinib modeled in the crystal structure of JAK1. A model of JAK1 complexed to upadacitinib is shown in blue. JAK2 (Protein Data Ban code: 2B7**a**) is overlaid in green (18). **b**. Chemical structure of (3S,4R)-3-ethyl-4-(3H-imidazo [1, 2-a] pyrrolo [2, 3-e] pyrazin-8-yl)-N-(2,2,2-trifluoroethyl) pyrrolidine-1-carboxamide (upadacitinib)

We developed structural hypotheses for obtaining JAK1 selectivity using internal and external JAK protein crystal structures as described previously [14]. When examining the amino acid homology/identity in the JAK family adenosine triphosphate (ATP) binding site, sequence conservation was high. JAK1 and JAK2 displayed ~ 85% sequence identity in the ATP binding pocket. Therefore, to achieve isoform selectivity, the design strategy focused on regions of structural differences in the active site as opposed to specific interactions with unique active site residues. Specifically, we hypothesized that the canonical glycine-rich loop assumed a “closed” backbone conformation in JAK1, in contrast to JAK2, due to amino acid sequence differences in the loop [14]. A primary design focus was to find an optimal group under the glycine-rich loop to exploit the differences in protein conformation between JAK1 and JAK2. Various groups were considered to stabilize a closed conformation of the loop. Fig. 1a shows a model of upadacitinib in JAK1 overlaid with the protein backbone of a previously reported x-ray structure of JAK2 (Protein Data Bank code: 2B7A) [15]. We predicted that the trifluoroethyl group optimally occupied the tight van der Waals interaction space under the glycine-rich loop to provide an induced fit into JAK1. The resulting structure of upadacitinib is shown in Fig. 1b.

### Upadacitinib is JAK1-selective and inhibits cytokines that contribute to the pathology of RA

To characterize the enzymatic activity of upadacitinib, we assessed potency and selectivity in biochemical assays utilizing recombinant human JAK kinases. The data is summarized in Table 1. Upadacitinib demonstrated activity against JAK1 (0.045 μM) and JAK2 (0.109 μM), with > 40 fold selectivity over JAK3 (2.1 μM) and 100 fold selectivity over TYK2 (4.7 μM) as compared to JAK1. Upadacitinib also demonstrated selectivity across a broad panel of 70+ kinases, with only Rock1 and Rock2 demonstrating IC_50_ values below 1 μM (Additional file 1: Table S1). To further characterize the mechanism of JAK1 inhibition of upadacitinib, we assessed JAK1 enzyme activity at varying concentrations of ATP. At all concentrations tested, the close agreement of the theoretical and experimental IC_50_ values confirmed that upadacitinib is an ATP competitive inhibitor (data not shown).

**Table 1:** In vitro potency of upadacitinib

Recombinant Human Kinase	IC50 nM		Fold selectivity vs. JAK1	
in Biochemical Assays^A^				
JAK1	47 +/− 6.1		1	
JAK2	120 +/− 29.6		2.5	
JAK3	2304 +/− 380.3		49	
TYK2	4690		100	
Engineered Cellular Assays				
Ba/F3 Cellular	IC50 nM		Fold selectivity vs. JAK1	
JAK1	14		1	
JAK2	593 +/− 118.7		42	
JAK3	1860 +/−207.2		133	
TYK2	2715 +/− 548.7		194	
Cytokine Signaling in Human Cells				
Cytokine	JAK	pSTAT	Cells	Human IC50 nM
IL-6	1	3	TF-1	11 +/− 1.3
IL-6	1	3	CD14+ whole blood	78 +/− 0.3
IL-6	1	3	CD3+ whole blood	207 +/− 9
OSM	1	3	TF-1	1.6 *N* = 2
Epo	2	5	UT7	649 +/− 41.2
IL-2	1/3	5	T-blasts	10 +/− 1.1
IL-15	1/3	5	T-blasts	22 +/−6.7
IFNγ	1/2	1	CD14+ monocytes	19
IL-4	1/3	6	HEKa ^B^	2
IL-13	1/TYK2	6	HEKa	4
IL-31	1/2	3	HEKa	3

^a^Enzyme reactions were conducted at 0.1 mM ATP. IC_50_ values represent the mean +/− SEM from at least three independent studies except where noted. ^B^ HEKa = human epithelial keratinocytes, adult

The JAK family selectivity of upadacitinib was confirmed in cellular assays. Due to the complexity of the cooperative nature of JAK kinases, we employed a set of engineered cell lines to understand the cellular potency and selectivity of upadacitinib on each individual kinase. As shown in Table 1, upadacitinib was > 40 fold selective for JAK1 (0.014 μM) as compared to JAK2 (0.593 μM). Upadacitinib also demonstrated selectivity against JAK3 (~ 130 fold) and TYK2 (~ 190 fold). The potency of upadacitinib was also assessed in physiologically relevant cellular systems. Consistent with the Ba/F3 cellular data, upadacitinib potently inhibited the JAK1 dependent cytokines IL-6, OSM, IL-2, and IFNγ, as measured by inhibition of STAT phosphorylation. This activity was ~ 60 fold more potent than the activity on erythropoietin signaling, a cytokine that depends exclusively upon JAK2 for signal transduction. We next measured inhibition of IL-6 signaling in human whole blood. The IC_50_ values for upadacitinib were 0.207 μM in the CD3+ T-cell population, and 0.078 μM in the CD14+ monocytic population. The reported IC_50_ values for tofactinib on IL-6 signaling in human whole blood are 0.367 μM and 0.406 μM for CD3+ T-cells and monocytes, respectively [12].

### Upadacitinib inhibits disease pathology in rat adjuvant induced arthritis

**Fig. 2:**
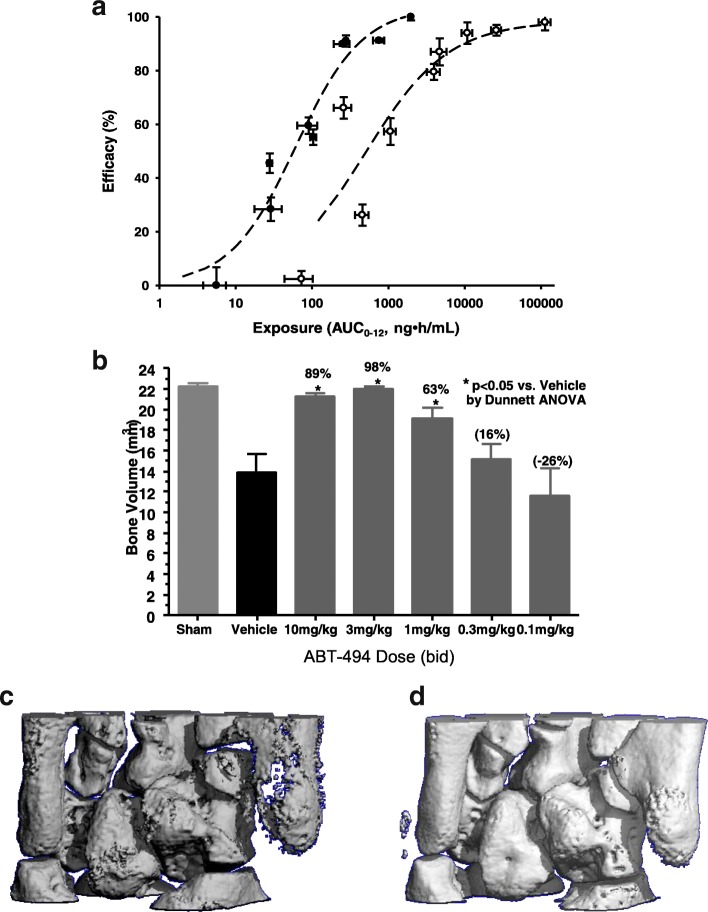
Preclinical efficacy of upadacitinib in rat adjuvant induced arthritis model. **a** Exposure–response relationships for upadacitinib (solid symbols) and tofacitinib (open symbols) in a rat adjuvant-induced arthritis model. **b** Quantitative effect of upadacitinib on bone erosion as assessed by micro CT scanning. **c** and **d** Representative micro CT scans of the tarsal regions of vehicle (**c**) and adjuvant-induced arthritis rats treated with upadacitinib (**d**)

To understand the effect on inflammation and the arthritis phenotype, we tested upadacitinib in the adjuvant induced arthritis model, an established preclinical model of RA. Orally administered upadacitinib was dosed at first signs of disease on day 7 and resulted in dose and exposure dependent reductions in paw swelling (Fig. 2a). On day 18 post disease induction, paws were harvested and bone destruction was measured by μCT. The normal course of AIA results in significant loss of bone volume that is dose dependently reduced with upadacitinib administration (Fig. 2b). Examples of destruction are shown from vehicle treated animals (Fig. 2c) with significant pitting and bone loss compared with the 10 mg/kg upadacitnib treated animals in which the surface of the bone was protected (Fig. 2d). Histological endpoints were also assessed in this study. Upadacitinib administration improved synovial hypertrophy, inflammation, cartilage damage and bone erosion at the 3 and 10 mg/kg dose groups (data not shown). Similar results were observed in the rat collagen induced arthritis (CIA) model, a second preclinical model of RA (data not shown). Tofacitinib was also tested in the AIA model and demonstrated dose-responsive efficacy, although the exposure-response curve was right shifted compared to upadacitinib (Fig. 2a). Efficacious concentrations were defined as the AUC_0–12_ drug concentration necessary to achieve 60% inhibition of paw swelling (AUC_60_). The rationale for using an AUC_60_ as a point of reference was based on the AUC exposure associated with the clinical dose of 10 mg BID of tofacitinib. This established a translational reference point for further analysis. The total efficacious drug exposure for upadacitinib was calculated to be 83 ng*hr./ml while this exposure was 1205 ng*hr./ml for tofacitinib. The increased in vivo potency of upadacitinib was expected based upon the difference in JAK1 cellular potency compared to tofacitinib.

### Upadacitinib spares reticulocyte deployment and NK cell count depletion relative to efficacy

**Fig. 3:**
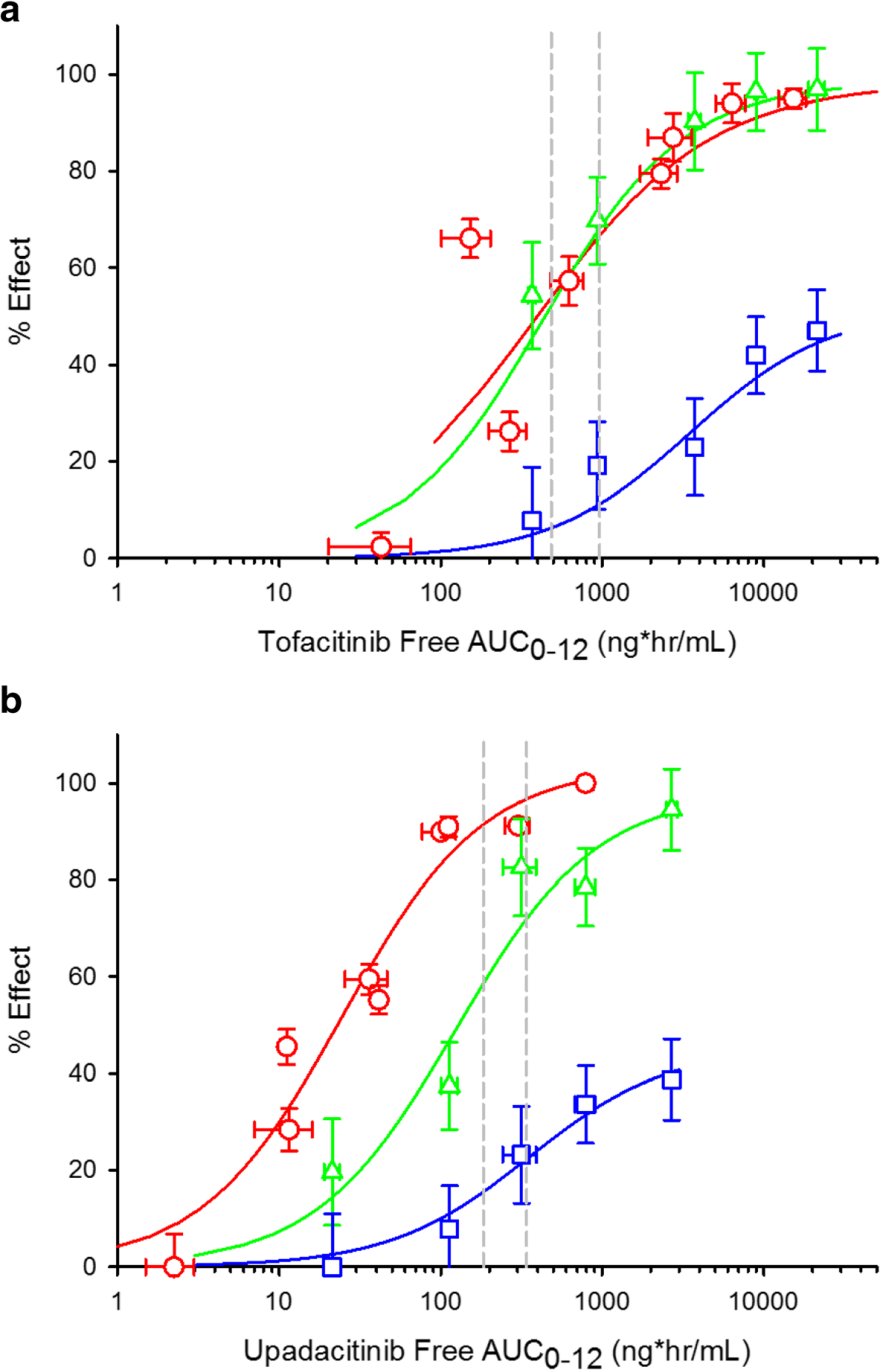
Rat in vivo selectivity of tofactinib and upadacitinib. **a**. Exposure response curves of tofacitinib on inhibition of paw swelling in rat AIA (red circles), CD3^−^/CD16^+^/CD56^+^ NK cell inhibition (green triangles), and inhibition of Epo induced reticulocyte deployment (blue squares). Dotted gray vertical lines represent equivalent clinical exposures for 5 mg and 10 mg tofacitinib. **b**. Exposure response curves of upadacitinib on inhibition of paw swelling in rat AIA (red circles), CD3^−^/CD16^+^/CD56^+^ NK cell inhibition (green triangles), and inhibition of Epo induced reticulocyte deployment (blue squares). Dotted gray vertical lines represent equivalent clinical exposures for 6 mg and 12 mg upadacitinib

We applied a variation of a method previously described [13] to determine the relative impact of upadacitinib and tofacitinib on inhibition of JAK2 dependent Epo receptor function. Naive rats were intravenously challenged with either PBS or 1000 IU of Epo on two consecutive days, and circulating reticulocytes were measured on day 4. Either upadacinitinb or tofactininib were dosed throughout and reticulocytes were quantified by flow cytometry. We also sought to determine the impact of upadacitinib and tofacitinib on common gamma chain signaling (JAK1/JAK3) in the form of circulating NK cell counts, given that these cells rely upon IL-15 for survival [16]. Naïve rats were dosed for 14 days with either upadacitinib or tofacitinib, and circulating CD3-/CD16+/CD56+ NK cells were quantified by flow cytometry. The results of the reticulocyte deployment, circulating NK cellcounts, and AIA efficacy experiments were plotted together to visualize the relative effects in relation to exposure (Fig. 3). Tofactinib decreases circulating NK cell numbers in an exposure dependent manner (AUC_60_ of 1230 ng*hr./ml) similar to the exposure range observed to inhibit paw swelling (AUC_60_ = 1205 ng*hr./ml). Tofacitinib reduced reticulocyte deployment in an exposure dependent manner reaching a maximal inhibition of > 40% at the highest concentration tested **(**Fig. 3a**)**. Upadacitinib decreases circulating NK cell numbers in an exposure dependent manner with an AUC_60_ = 480 ng*hr./ml, ~ 5 fold less potent than the concentration of drug required to inhibit paw swelling (AUC_60_ = 83 ng*hr./ml). Reticulocyte deployment was also dose-responsively reduced and reached a maximal inhibition of ~ 40% (Fig. 3b). At the clinical AUC exposures associated with 10 mg BID of tofacitinib, reduction in paw swelling was ~ 60% and there is a clear overlap with NK cell depletion (Fig. 3a). Utilizing exposures for 6 mg BID and 12 mg BID clinical doses of upadacitinib, reduction in paw swelling was > 90%, with a distinct separation from NK cell depletion (Fig. 3b).

**Fig. 4:**
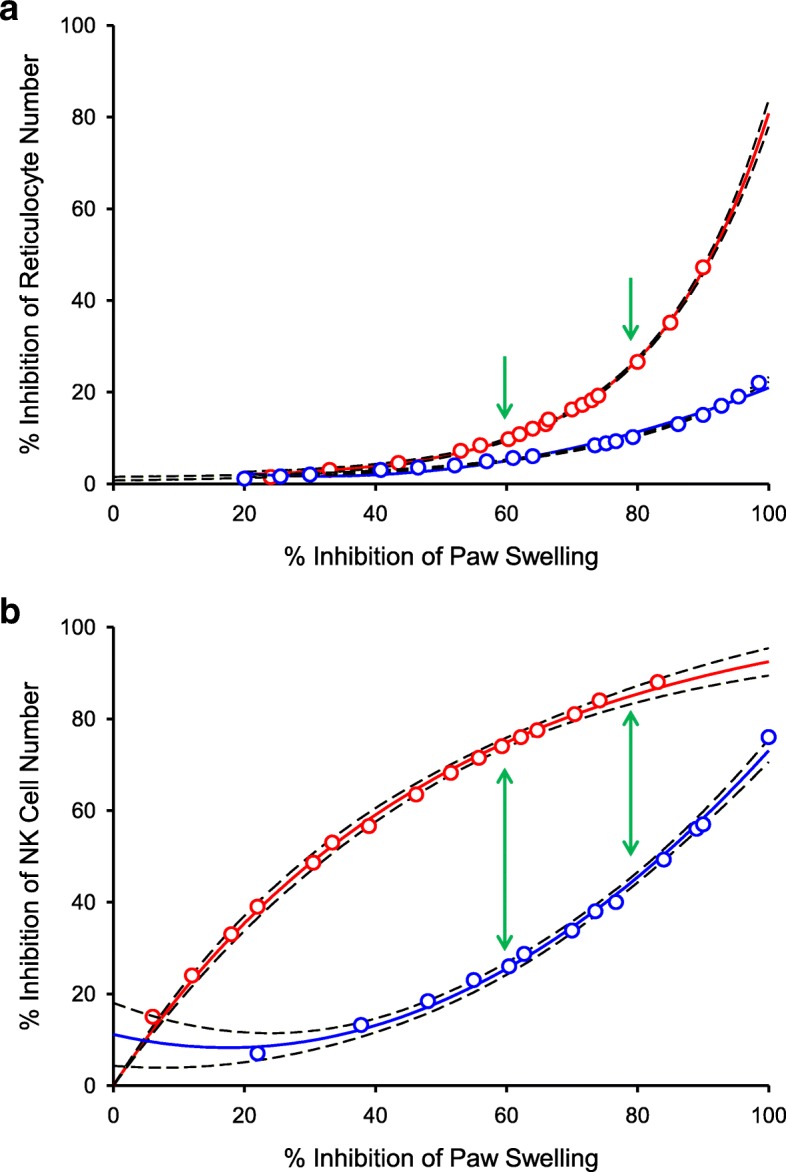
**a** Effect on reticulocyte inhibition compared with inhibition of paw swelling in rat AIA for tofacitinib (red circles) and upadacitinib (blue circles) Green arrows denote 60 and 80% inhibition of paw swelling. **b** Effect on NK cell inhibition compared with inhibition of paw swelling in rat AIA for tofacitinib (red circles) and upadacitinib (blue circles). Green arrows denote 60 and 80% inhibition of paw swelling. Individual animal values are graphed with 95% confidence intervals represented with black dotted lines

The reticulocyte (Fig. 4a) and NK cell (Fig. 4b) data were replotted versus % inhibition of paw swelling in the AIA model to directly compare the inhibitory effects of tofacitinib and upadacitinib as a function of disease efficacy. The relative impact on reticulocytes is similar between tofacitinib and upadacitinib at the lower efficacious range, but at the higher efficacious range (> 60% of paw swelling), the differential effect becomes much more pronounced (Fig. 4a). Likewise, there is a clear differential effect on circulating NK cell counts. At AUC_60_, there is a 70% decrease in circulating NK cells upon tofacitinib treatment, while upadacitinib treatment results in a 25% decrease (Fig. 4b).

### Upadacitinib spares common gamma chain signaling relative to IL-6 signaling in healthy volunteers

**Fig. 5:**
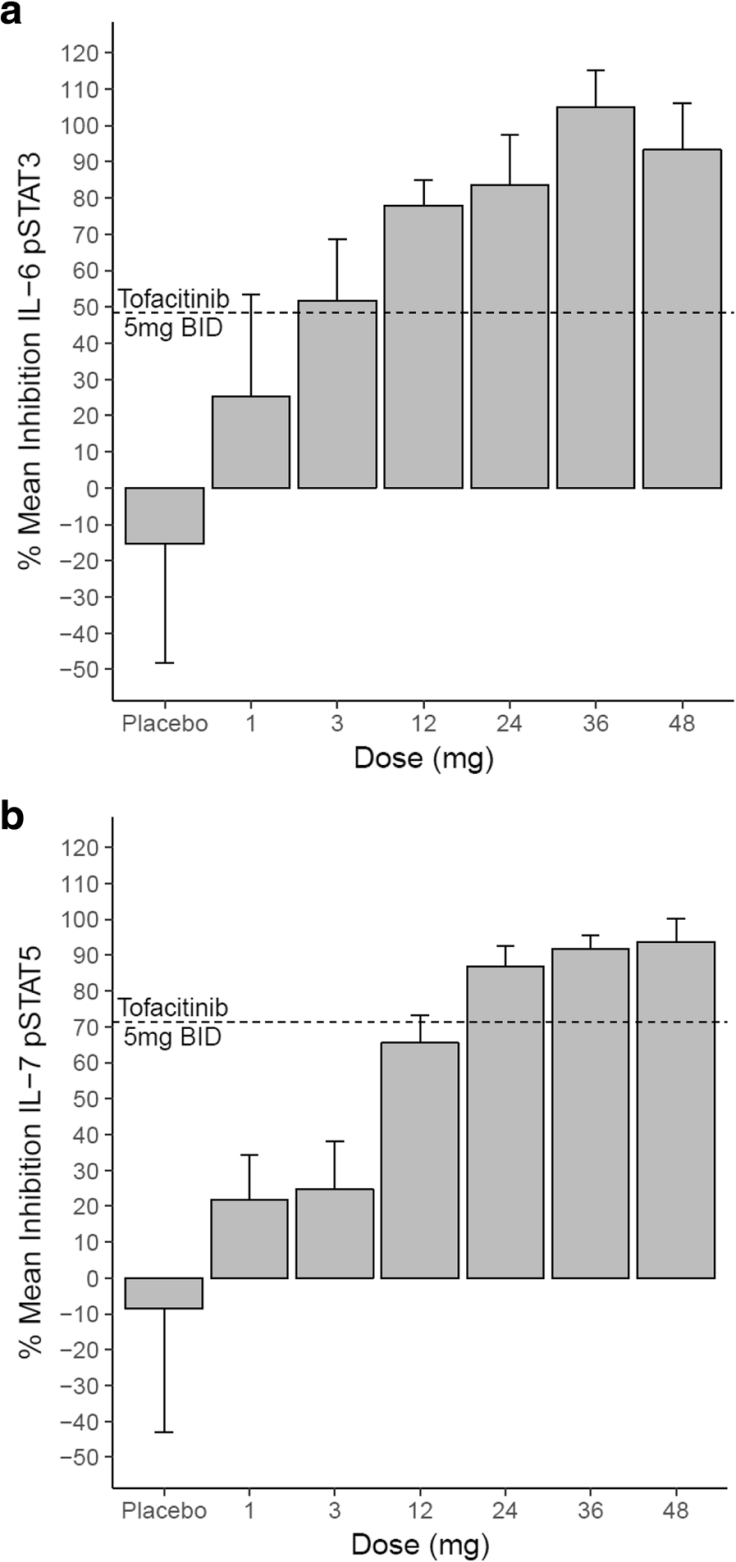
Ex vivo stimulation of whole blood from upadacitinib or placebo dosed healthy volunteers. Whole blood was drawn 1 h post upadacitinib or tofacitinib dose and stimulated with IL-6 (A) or IL-7 (B)

To confirm the preclinical selectivity of upadacitinib in a clinical setting, ex vivo cytokine stimulation assays were performed in the whole blood of healthy volunteers dosed with 1, 3, 12, 24, 36, or 48 mg of upadacitinib, or with 5 mg of tofacitinib. At 1 h post dose, blood was drawn and stimulated with IL-6 or IL-7 to assess the impact of upadacitinib on these signaling pathways. Inhibition of downstream STAT phosphorylation (STAT3 and STAT5) was assessed by flow cytometry (Fig. 5). JAK1 mediated IL-6 induced pSTAT3 was inhibited ~ 50% at the 3 mg dose of upadacitinib, equivalent to the level of inhibition seen with 5 mg of tofacitinib. Increasing doses of upadacitinib demonstrated concomitant increases in pSTAT3 inhibition before reaching maximal inhibition at 36 mg. For the purpose of evaluating JAK1/3 potency in vivo, activity against common γ chain signaling was assessed using IL-7 driven pSTAT5. In this case, 12 mg of upadacitinib was necessary to inhibit pSTAT5 to the same degree as 5 mg tofacitinib (~ 70%).

## Discussion

Upadacintinib (ABT-494) is a selective JAK1 inhibitor that was designed to address the hypothesis of whether greater JAK1 selectivity over other JAK family members would translate clinically to an improved benefit:risk profile compared to tofactinib, the first approved JAK inhibitor for the treatment of RA. Several other JAK inhibitors are currently under clinical development for the treatment of RA, including baricitinib (INCB28050) and peficitinib (ASP015K). Filgotinib, which has been reported to be a specific JAK1 inhibitor, is also currently in clinical trials for RA. Here, we have shown the in vitro JAK1 selectivity profile of upadacitinib, the in vivo physiological consequences of that selectivity, and the clinical translation of the JAK1 selectivity in healthy volunteers dosed with upadacitinib.

In the discovery and development of upadacitinib, JAK1 selectivity was thought to be important based on a number of factors. The efficacy achieved by tofacitinib in RA clearly demonstrated the potential of JAK inhibition in modulating disease. However, dose limiting side effects were associated with the improved efficacy seen at higher doses [11]. The nature of the side effects, coupled with the selectivity profile of tofacitinib, provided clues as to the potential mechanisms driving these toxicities. For example, a reduction in hemoglobin levels with tofacitinib may be driven by inhibition of JAK2, an essential driver of erythropoiesis. Increased infection risk at higher doses of tofacitinib could also be driven by JAK3 inhibition and subsequent downstream blockade of IL-15 survival signaling in NK cells. By improving JAK1 selectivity, the goal was to inhibit JAK1 dependent processes that drive pathology, such as IL-6 and IFNγ signaling, while sparing dose limiting adverse events possibly associated with JAK2 and JAK3 inhibition and thereby improving the overall benefit:risk profile.

Structural understandings of JAK1 and JAK2 led to the development of a selective JAK1 inhibitor that can be tested in clinic. The canonical glycine-rich loop in JAK1 assumes a “closed” conformation compared to JAK2 due to amino acid differences within the loop. A trifluoromethyl group within upadacitinib was designed to occupy van der Waals interaction space in the loop to provide an induced fit into JAK1 thereby confering selectivity (Fig. 1). The selectivity was confirmed in both biochemical and cellular assay systems (Table 1). Biochemically, upadacitinib is most potent on JAK1compared to other family members (JAK1 > JAK2 > JAK3 > TYK2). We used engineered cell lines to study the potency of upadacitinib on JAK family members in isolation, and cellular assays with physiologically relevant stimuli to define the effects in a native setting. While there was only a modest 2.5 fold JAK1 selectivity over JAK2 in biochemical assays, cellular data indicates that the JAK1 selectivity of upadacitinib compared to JAK2 is greater than that demonstrated in biochemical assays. STAT3 phosphorylation driven by IL-6 and IFNγ is effectively blocked by upadacitinib in the low nM range, while Epo-driven STAT5 phosphorylation is only modulated at a ~ 60 fold higher concentration. We hypothesized this difference would allow for full efficacious potency against inflammatory cytokines driving disease, while sparing potentially adverse effects from blockade of Epo signaling. Interrogating the cellular potencies of JAK3 and TYK2 is problematic due to the interdependency of these family members with JAK1 in signaling processes. To address this, we employed a set of engineered cell lines to gain a greater understanding of the potency of upadacitinib on these kinases in isolation as has been used with other inhibitors of the class [17]. For example, upadacitinib potently inhibits IL-2 signaling (13 nM), a common γ chain cytokine that utilizes both JAK1 and JAK3. The low potency observed in the TEL-JAK3 assay (1820 nM) suggests the activity of upadacitinib is driven almost entirely by activity against JAK1. For both JAK3 and TYK2 upadacitinib is > 100 fold more selective for JAK1.

We next sought to extend the in vitro findings and determine the in vivo physiological consequence of JAK1 selectivity in a number of animal model systems. The ability of upadacitinib to ameliorate arthritis was tested in the rat adjuvant induced arthritis model, a well-established model of RA. This model has been shown to have significant expression of IL-6 and IFN γ [18], both of which are JAK1 mediated cytokines. Upadacitinib administration suppressed paw swelling and bone destruction in a dose and exposure dependent manner to a similar degree as tofacitinib (Fig. 2). The efficacious exposure of upadacitinib was significantly more potent in the AIA model as compared to tofacitinib consistent with the in vitro potency on IL-6 pSTAT3 inhibition.

Tofacitinib suppressed NK cell counts with equal potency as inhibition of paw swelling in the AIA model. This is consistent with the in vitro potency that shows that tofacitinib inhibits JAK1 and JAK3 to a similar extent. After upadacitinib administration, the inhibition of NK cell counts was 5-fold less sensitive than inhibition of paw swelling (Fig. 3), resulting in an overall reduced effect against NK cell suppression per unit efficacy for upadacitinib. This difference may be in part attributable to the weak JAK3 potency of upadacitinib, whereas tofacitinib has been reported to potently inhibit both JAK3 and JAK1 [12]. It can be anticipated that inhibition of both JAK1 and JAK3 could additively or synergistically effect common γ chain cytokines.

We used an in vivo model of reticulocyte deployment as a way to evaluate the effect of each inhibitor on JAK2. The difference between tofacitinib and upadacitinib on reticulocyte counts was most pronounced at higher efficacious ranges of AIA model (Fig. 4a). At 60% inhibition of paw swelling, there was minimal difference between tofacitinib and upadacitinib on reticulocyte number but at 80% inhibition of paw swelling, tofacitinib inhibited deployment of reticulocytes to a greater extent than upadacitinib. The in vivo physiological parameters of reduction in paw swelling as compared to inhibition of reticulocytes counts and circulating NK cell numbers can be explained by the relative cellular JAK family selectivity profiles of upadacitinib and tofacitinib. Because the cellular data was predictive of the in vivo PK/PD effect, we used this criteria to define the JAK family selectivity of upadacitinib.

The preclinical selectivity data was confirmed in healthy volunteeers dosed with upadacitinib. There was a clear separation between inhibition of IL-6 and L-7 signaling up to 12 mg, reflective of the in vitro JAK1 selectivity of upadacitinib. The data from these ex vivo stimulation assays conducted in phase 1 studies was instrumental in designing optimal doses for phase 2 proof of concept studies in RA. Two phase 2 studies evaluating the efficacy of upadacitinib in RA patients have been recently published [19, 20]. The data from these studies allowed us to evaluate the translation of the preclinical selectivity data and the pharmacodynamics effects in healthy volunteers to the effect observed in RA patients treated with upadacitinib. Upadacitinib treatment in patients with either inadequate response to methotrexate (MTX-IR, BALANCE II) or inadequate response to anti-TNF therapy (BALANCE I) showed rapid, dose-dependent improvement in signs and symptoms. In BALANCE I, mean hemoglobin values remained stable at the 3 mg and 6 mg doses and decreased dose dependently at higher doses [19]. Dose dependent changes in hemoglobin were also observed in the BALANCE II but the values remained in the normal range [20]. This data is consistent with separation of JAK1 from JAK2 activity at lower doses and potential increases in engagement of JAK2 at higher concentrations. Similarly for JAK1/JAK3 engagement, NK cell numbers dose dependently decreased in doses ≥6 mg BID. The significance of NK cell reductions on infection and immune surveillance remains unclear. The physiologic selectivity as demonstrated in the preclinical models translates well in patients with RA as the minimally efficacious dose from the preclinical studies was predicted to be 3 mg BID where significant effects on signs and symptoms were observed with minimal effect on either hemoglobin or NK cell numbers.

Given the selectivity profile of upadacitinib, potential pathogenic drivers in RA such as GM-SCF, IL-2, IL-9, IL-12, and IL-23, mediated by JAK2, JAK3, and TYK2 will be less impacted at efficacious concentrations. It is currently unknown how these pathways contribute to disease and whether these pathways are orthogonal or overlapping to JAK1-mediated drivers. However, the efficacy demonstrated with upadacitinib in Phase 2 studies in RA suggests that the selectivity profile is sufficient to inhibit disease to a significant extent.

## Conclusion

The in vitro and in vivo data shown here demonstrate that upadacitinib is a JAK1 selective inhibitor. This profile has shown promise in phase 2 studies in patients with RA. Phase 3 studies will be needed to allow further characterization of the benefit:risk profile of upadacitinib in the treatment of RA.

## Additional file

Additional file 1: **Table S1.** Upadacitinib Kinome Selectivity. Of the kinases in the panel, 14 kinases have an IC_50_ below 10 μM, but only 2 non-JAK kinases have IC_50_ values below 1 μM (Rock1 at 0.92 μM and Rock2 at 0.43 μM). JAK activity assays using isolated kinase domains in the presence of 0.1 mM ATP and trFRET kinome profiling were conducted as described previously (17). ATP, adenosine triphosphate; HTRF, homogenous trFRET; IC_50_, concentration producing 50% inhibition; JAK, Janus kinase; trFRET, time-resolved fluorescence energy transfer. (DOCX 22 kb)

## Abbreviations

AIA: Adjuvant induced arthritis
ATP: Adenosine triphosphate
EPO: Erythropoietin
IC_50_: Inhibitory concentration at which there is a 50% effect
JAK: Janus kinase
PK/PD: Pharmacokinetic/pharmacodynamics effect
RA: Rheumatoid arthritis
STAT: signal transducer and activator of transcription protein
TYK: Tyrosine kinase
