# Supplementary material for: In vitro and in vivo characterization of the JAK1 selectivity of upadacitinib (ABT-494)
Source: BMC Rheumatol. 2018 Aug 28;2:23. doi: 10.1186/s41927-018-0031-x (PMC6390583; doi:10.1186/s41927-018-0031-x)
Supplement: Supplementary file 1 — Table S1. Upadacitinib Kinome Selectivity. Of the kinases in the panel, 14 kinases have an IC50 below 10 μM, but only 2 non-JAK kinases have IC50 values below 1 μM (Rock1 at 0.92 μM and Rock2 at 0.43 μM). JAK activity assays using isolated kinase domains in the presence of 0.1 mM ATP and trFRET kinome profiling were conducted as described previously (17). ATP, adenosine triphosphate; HTRF, homogenous trFRET; IC50, concentration producing 50% inhibition; JAK, Janus kinase; trFRET, time-resolved fluorescence energy transfer. (DOCX 22 kb) [file 41927_2018_31_MOESM1_ESM.docx]

SUPPLEMENTARY INFORMATION

Supplementary Table 1. Upadacitinib Kinome Selectivity

Of the kinases in the panel, 14 kinases have an IC_50_ below 10 μM, but only 2 non-JAK kinases have IC_50_ values below 1 μM (Rock1 at 0.92 μM and Rock2 at 0.43 μM).

JAK activity assays using isolated kinase domains in the presence of 0.1 mM ATP and trFRET kinome profiling were conducted as described previously ([17](#_ENREF_17)).

| **Kinase** | **Assay** | **[ATP] (mM)** | **Average IC_50_ (μM)** |
| --- | --- | --- | --- |
| JAK1 | HTRF activity | 0.1, 0.001 | 0.043, <0.0032 |
| JAK2 | HTRF activity | 0.1, 0.001 | 0.12, <0.0032 |
| JAK2 | trFRET binding | - | 0.008 |
| JAK3 | HTRF activity | 0.1, 0.001 | 2.1, 0.054 |
| JAK3 | trFRET binding | - | 0.068 |
| TYK2 | HTRF activity | 0.1, 0.001 | 0.055, 4.7 |
| ABL | trFRET binding | - | >10 |
| ACVR1 | trFRET binding | - | >10 |
| Akt1 | trFRET binding | - | >10 |
| ALK | trFRET binding | - | >5.5 |
| AMPK | trFRET binding | - | >4.5 |
| Aurora 1 | trFRET binding | - | >5.5 |
| Aurora 2 | trFRET binding | - | 4.5 |
| BRAF | trFRET binding | - | >10 |
| BTK | trFRET binding | - | >10 |
| CAMK1D | trFRET binding | - | 2.3 |
| CAMK2A | trFRET binding | - | >10 |
| CAMKK2 | trFRET binding | - | >10 |
| CDK11 | trFRET binding | - | >10 |
| CDK2 | trFRET binding | - | >10 |
| CDK7/Cyclin H/Mat1 | trFRET binding | - | >10 |
| CDK8/Cyclin C | trFRET binding | - | >10 |
| CDK9/Cyclin K | trFRET binding | - | >10 |
| CLK2 | trFRET binding | - | >10 |
| Ck1alpha1 | trFRET binding | - | >10 |
| cMET | trFRET binding | - | >10 |
| CSF1R | trFRET binding | - | >10 |
| DDR1 | trFRET binding | - | >10 |
| DYRK1A | trFRET binding | - | >10 |
| DYRK1B | trFRET binding | - | >10 |
| EGFR | trFRET binding | - | >10 |
| Erk2 | trFRET binding | - | >10 |
| FAK | trFRET binding | - | >10 |
| FGFR1 | trFRET binding | - | 4.8 |
| Flt1 | trFRET binding | - | >10 |
| Fyn | trFRET binding | - | >10 |
| GRK5 | trFRET binding | - | >7.9 |
| Gsk3a | trFRET binding | - | >10 |
| Gsk3b | trFRET binding | - | >10 |
| IGF1R | trFRET binding | - | >10 |
| IKKE | trFRET binding | - | >10 |
| InsR | trFRET binding | - | >10 |
| JNK1 | trFRET binding | - | >10 |
| JNK2 | trFRET binding | - | >10 |
| KDR | trFRET binding | - | >1.5 |
| LCK | trFRET binding | - | >10 |
| LTK | trFRET binding | - | 2.7 |
| MAP2K3 | trFRET binding | - | >10 |
| MAP3K10 | trFRET binding | - | >10 |
| MAP4K2 | trFRET binding | - | >10 |
| MAP4K4 | trFRET binding | - | >10 |
| MEK1 | trFRET binding | - | >10 |
| MEK2 | trFRET binding | - | >10 |
| MST1 | trFRET binding | - | >10 |
| Nek2 | trFRET binding | - | >10 |
| PAK4KD | trFRET binding | - | >10 |
| PDGFRA V561D | trFRET binding | - | >10 |
| PDGFRB | trFRET binding | - | >10 |
| Pim1 | trFRET binding | - | >10 |
| Pim2 | trFRET binding | - | >10 |
| PKA | trFRET binding | - | >10 |
| PKCtheta | trFRET binding | - | 5.1 |
| PKCzeta | trFRET binding | - | >10 |
| PKG1A | trFRET binding | - | >10 |
| Plk3 | trFRET binding | - | >10 |
| Prkcn | trFRET binding | - | >10 |
| p38 alpha | trFRET binding | - | >10 |
| RET | trFRET binding | - | 2.4 |
| Rock1 | trFRET binding | - | 0.92 |
| Rock2 | trFRET binding | - | 0.42 |
| Rsk2 | trFRET binding | - | >9.6 |
| SGK1 | trFRET binding | - | >10 |
| Src | trFRET binding | - | >10 |
| STK16 | trFRET binding | - | 9.7 |
| STK33 | trFRET binding | - | >10 |
| Syk | trFRET binding | - | >10 |
| TAOK2 | trFRET binding | - | >10 |
| TBK1 | trFRET binding | - | >10 |
| TNK2 | trFRET binding | - | 5.1 |
| TrkA | trFRET binding | - | >10 |
| TrkB | trFRET binding | - | >10 |
| TrkC | trFRET binding | - | >10 |
| TTK | trFRET binding | - | >10 |
| TYRO3 | trFRET binding | - | >10 |
| Wee1 | trFRET binding | - | >10 |
| Zipk | trFRET binding | - | >10 |

ATP, adenosine triphosphate; HTRF, homogenous trFRET; IC_50_, concentration producing 50% inhibition; JAK, Janus kinase; trFRET, time-resolved fluorescence energy transfer.
